# Gastrointestinal bleeding after endoscopic mucosal resection in a case of Peutz–Jeghers syndrome with hypofibrinogenemia: A case report

**DOI:** 10.3389/fped.2022.961501

**Published:** 2022-10-05

**Authors:** Toshihiko Kakiuchi, Hironobu Takedomi, Takashi Akutagawa, Nanae Tsuruoka, Yasuhisa Sakata, Muneaki Matsuo

**Affiliations:** ^1^ Department of Pediatrics, Faculty of Medicine, Saga University, Saga, Japan; ^2^ Division of Gastroenterology, Department of Internal Medicine, Faculty of Medicine, Saga University, Saga, Japan

**Keywords:** Peutz–Jeghers syndrome, hypofibrinogenemia, endoscopic mucosal resection, complications, gastrointestinal hemorrhage

## Abstract

**Backgroud:**

Peutz–Jegers syndrome (PJS) is an autosomal dominant hereditary disorder characterized by hamartomatous polyposis of the entire gastrointestinal tract. Fibrinogen (Fbg) is synthesized by the liver, and hypofibrinogenemia is often asymptomatic and manifests with bleeding after trauma or invasive surgical procedures. Here, we present a case of a pediatric patient with PJS and hypofibrinogenemia who manifested with gastrointestinal bleeding after endoscopic mucosal resection (EMR) of small intestinal polyps.

**Case Presentation:**

An 11-year-old boy with PJS was referred to our hospital. Since his mother was diagnosed with PJS, with black pigments being observed on his lips, mouth, and limbs, he underwent upper and lower gastrointestinal endoscopy at the age of 8 years at a previous hospital. EMR for duodenal polyp was performed, and the pathological findings were consistent with hamartoma. His Fbg level was 117 mg/dl at the time, with no post-bleeding being detected after EMR. The small intestine was not assessed at the prior facility and was left neglected for three years. At our hospital, small intestine fluoroscopy was performed and revealed a polyp in the jejunum, and abdominal computed tomography showed two polyps and intussusception. On double-balloon enteroscopy, the resected polyps were hamartoma with diameters of 20 and 30 mm. The patient’s Fbg level was 107 mg/dl. The day after EMR, he had melena and black stools. He was diagnosed with post-EMR bleeding and started to stop eating, and hemostatic agents were given. His hemoglobin level dropped to 9.2 g/dl the next day. Genetic testing for congenital Fbg deficiency revealed a heterozygous pathogenic variant in *fibrinogen gamma chain* Exon 10. Therefore, he was diagnosed with concurrent hypofibrinogenemia and PJS.

**Conclusion:**

To the best of our knowledge, this is the first reported case with concurrent PJS and hypofibrinogenemia. In patients with PJS, hypofibrinogenemia should be considered as one of the risk factors of postoperative bleeding during polypectomy, and appropriate prophylactic measures should be taken.

## Introduction

Peutz–Jegers syndrome (PJS) is an autosomal dominant hereditary disorder characterized by hamartomatous polyposis of the entire gastrointestinal tract excluding the esophagus and pigmented spots on the lips, oral cavity, fingertip skin, and mucous membranes (1). Most cases (>90%) appear to be caused by a germline mutation of the serine/threonine kinase 11 (*STK11/LKB1*) tumor suppressor gene, which is located on chromosome 19p13 (2, 3). This type of hamartomatous polyps found in PJS, known as Peutz–Jeghers polyps, is characterized by hamartomatous hyperplasia of the mucosal epithelium along with dendritic growth of smooth muscle fiber bundles from the muscularis mucosae (4). Peutz–Jeghers polyps cause symptoms, such as black stools, anemia, abdominal pain, and vomiting, while enlarged polyps often cause intussusception and necessitate surgical treatment (5).

Fibrinogen (Fbg) is one of the most abundant plasma proteins that is synthesized by the liver (6) and circulates in the blood, with a normal level ranging from 150 to 350 mg/dl (7). Fbg disorders are classified into three categories: afibrinogenemia with complete absence of Fbg; hypofibrinogenemia with decreased plasma Fbg levels; and dysfibrinogenemia with normal Fbg levels (150–350 mg/dl) and low functional activity (8). Congenital hypofibrinogenemia is caused by a variety of inherited gene defects (9). Although patients with afibrinogenemia are manifested by the umbilical cord, mucosal, intracerebral, and/or intraabdominal bleeding, those with hypofibrinogenemia are often asymptomatic and manifest with bleeding after trauma or invasive surgical procedures (7).

To the best of our knowledge, no case of concurrent PJS and hypofibrinogenemia has been reported. In this report, we present a pediatric case of PJS with hypofibrinogenemia who bled after endoscopic mucosal resection (EMR) of small intestinal polyps.

## Case report

**Figure 1:**
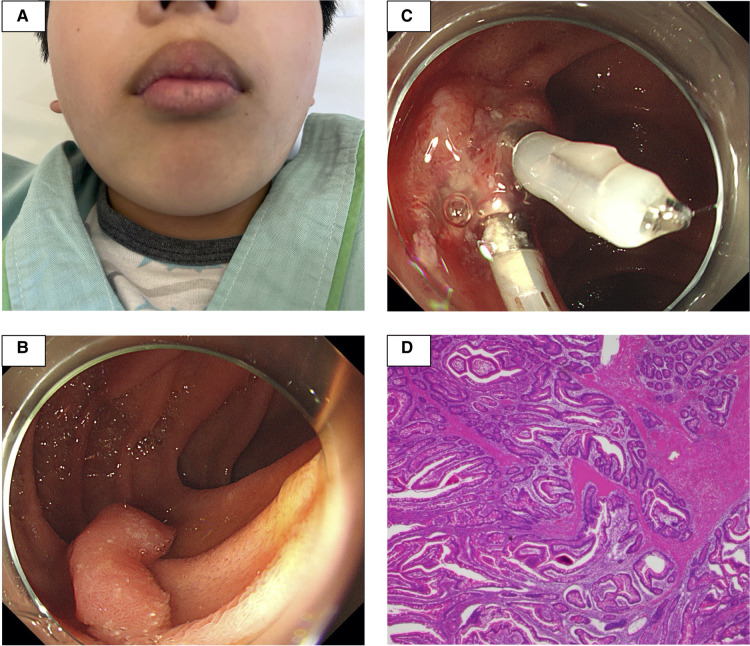
The patient had black pigmented spots on his lips (**A**). At a previous hospital, esophagogastroduodenoscopy revealed a 20 mm stalked polyp in the descending portion of the duodenum. After endoscopic mucosal resection, hemostasis was performed at the excision site with two clips (**B,C**). The histopathological examination revealed dendritic growth and proliferation of the muscularis mucosae and hyperplasia of the small intestinal epithelium, all of which are consistent with Peutz–Jeghers syndrome (**D**).

A 9-year-old boy was admitted to a previous hospital with fever and systemic purpura due to parvovirus B19 infection. He noticed black pigmented spots on his lips, buccal mucosa, fingertips, and soles of his feet at the time (Figure 1A). He was diagnosed with PJS in the same way as his mother, who had a bowel resection due to intussusception caused by a small-bowel polyp and was definitely diagnosed with PJS. The results of his laboratory tests were as follows: the white blood cell (WBC) count was 2,500/*μ*l (normal range: 7,000–15,000), the hemoglobin level was 13.1 g/dl (normal range: 13.7–16.8), the platelet count was 184,000 cells/*µ*l, the prothrombin time-international normalized ratio (PT-INR) was 1.13 (normal range: 0.85–1.15), the activated partial thromboplastin time (APTT) was 42.3 s (normal range: 20–40), the Fbg level was 126 mg/dl (normal range: 150–350) and the Fbg/fibrin degradation products (FDP) level was 0.6 *μ*g/ml (normal range: <5). Although the Fbg level was low, his other blood coagulation profile was normal. Furthermore, his laboratory test results for liver and renal function were normal. His symptoms (fever and leukopenia) were presumed to be due to parvovirus B19 infection because the results for parvovirus B19-immunoglobulin M antigen were positive.

Three months later, he was admitted to the hospital for esophagogastroduodenoscopy (EGD) and total colonoscopy to assess PJS gastrointestinal polyps. The results of his laboratory tests at the time were as follows: The WBC count was 7,000/*μ*l, the platelet count was 305,000 cells/*µ*l, the PT-INR was 1.00, the APTT was 31.2 s, the Fbg level was 117 mg/dl and the FDP level was 0.6 *μ*g/ml. Flat polyps of less than 10 mm in diameter were detected sporadically in the stomach and colon, and a 20 mm stalked polyp was found in the descending portion of the duodenum. Since no obvious bleeding symptoms were observed in daily life, EMR for duodenal polyps was performed (Figure 1B), and hemostasis was performed at the excision site with two clips (Figure 1C). Despite being continuously monitored for a month following the operation, no obvious postoperative bleeding was observed. The histopathological examination revealed dendritic growth and proliferation of the muscularis mucosae as well as hyperplasia of the small intestinal epithelium, all of which are consistent with PJS (Figure 1D).

**Figure 2:**
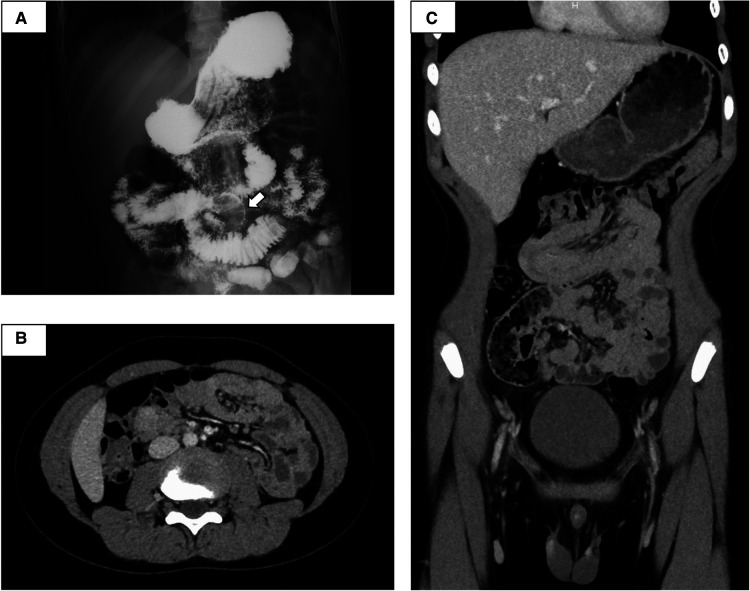
Small intestine fluoroscopy revealed a polyp in the jejunum (**A**), and abdominal computed tomography showed two polyps and intussusception (**B,C**). White arrow, polyp.

**Figure 3:**
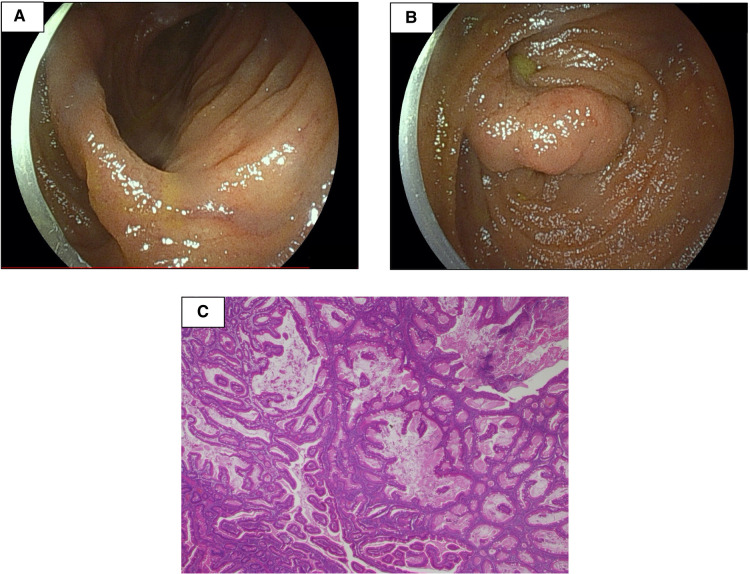
Double-balloon enteroscopy revealed two polyps with diameters of 20 and 30 mm (**A,B**). The pathological findings were consistent with hamartoma (**C**).

**Figure 4:**
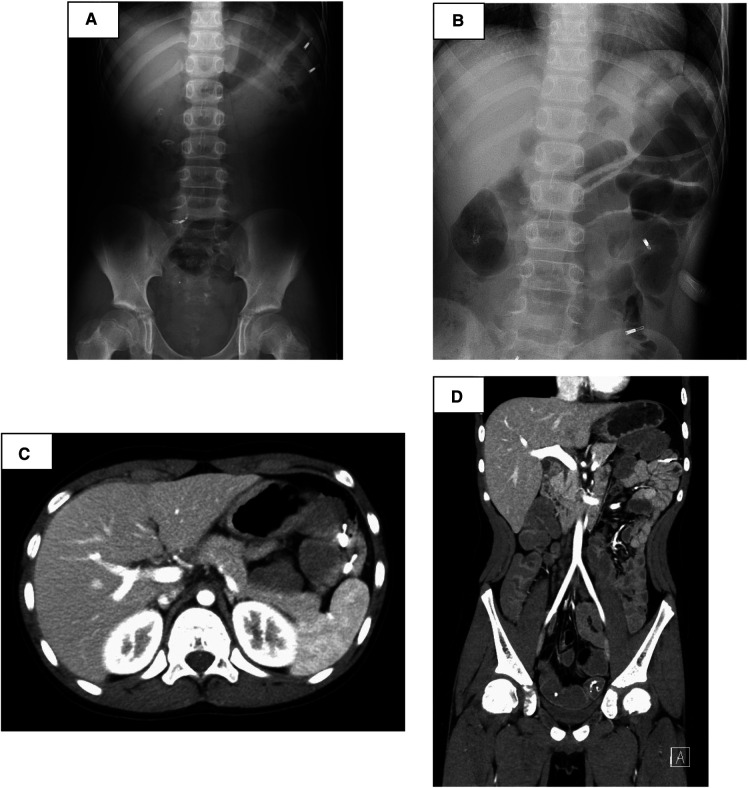
After endoscopic mucosal resection—suspected post-bleeding, abdominal x-rays (**A**) and enhanced abdominal computed tomography scan revealed that the position of the clips had not changed from the position immediately after EMR (**B**), and there were no signs of gastrointestinal perforation or arterial bleeding (**C,D**).

The small intestine was not assessed at the previous facility and was neglected for 3 years because evaluating the small intestine was difficult at that facility. Although the patient had no abdominal symptoms, he was referred to our hospital at the age of 11 years for the evaluation of small intestine. Small intestine fluoroscopy was conducted and revealed a polyp in the jejunum (Figure 2A), and abdominal computed tomography (CT) scan showed two polyps and intussusception (Figures 2B,C), but no gastrointestinal symptoms were experienced. Since no obvious bleeding symptoms in daily life and bleeding after EMR for duodenal polyps were observed, he proceeded to the next step without workup for low Fbg level at this time. On double-balloon enteroscopy, the resected polyps were hamartoma with diameters of 20 and 30 mm (Figures 3A,B). Hemostasis was performed at the excision site with one clip following EMR for small intestinal polyps because the operability of the endoscope was poor. The pathological findings indicated a hamartoma (Figure 3C). The Fbg level was 107 mg/dl, with a normal coagulation profile. He manifested with melena and black stools on the day after EMR (approximately 20 h after the EMR). The results of his laboratory tests were as follows: the hemoglobin level was 13.1 g/dl, the platelet count was 212,000 cells/*µ*l, and the Fbg level was 74 mg/dl. Abdominal x-rays (Figure 4A) and enhanced abdominal CT scan at the time revealed that the position of the clips had not changed from the position immediately after EMR (Figure 4B), and there were no signs of gastrointestinal perforation or arterial bleeding (Figures 4C,D). As he had no history or family history of thrombosis and other thrombotic risk factors, such as pregnancy and/or immobilization (10), he was eventually diagnosed with post-EMR bleeding. He stopped eating, and hemostatic agents (carbazochrome sodium sulfonate hydrate for 75 mg/day and tranexamic acid for 750 mg/day) were administered for 5 days. The next day's stool was only black with no melena. His hemoglobin level dropped to 9.2 g/dl but recovered to 9.6 g/dl the next day with no need for blood transfusions. As the low Fbg level before EMR was thought to a cause of gastrointestinal bleeding, genetic analysis of peripheral blood for congenital Fbg deficiency was performed, and it revealed a heterozygous pathogenic variant in Exon 10 of the *fibrinogen gamma chain* (*FGG*) gene (OMIM: 202400) [c.769 G > T (*p*.Glu257Ter)]. However, mutations in the STK11/LKB gene (the causative gene of PJS) were not evaluated as the diagnosis of PJS was confirmed by the patient's clinical symptoms and family history.

## Discussion

To the best of our knowledge, this is the first report on a case with concurrent PJS and hypofibrinogenemia. The present case suggests that EMR in cases with PJS and congenital bleeding risk, such as hypofibrinogenemia, necessitates careful preparation and monitoring.

Approximately 400 families of congenital fibrinogen abnormalities have been recorded worldwide (11), including quantitative abnormalities, such as afibrinogenemia and hypofibrinogenemia, and qualitative abnormalities, such as dysfibrinogenemia (12). In afibrinogenemia and hypofibrinogenemia, various genetic aberrations are detected in homozygotes and heterozygotes, with Fbg being deficient in plasma in homozygotes, and reduced in heterozygotes. However, most Fbg dysfunctions are caused by missense mutations in heterozygotes, which alter the three-dimensional structure of Fbg and impair the blood coagulation process (10, 13). The present case was diagnosed with hypofibrinogenemia based on the presence of a heterozygous pathogenic variant in Exon 10 of *FGG*, as previously reported (14). *STK11*, which is involved in PJS, is located on chromosome 19p13.3, whereas *FGG* is located on chromosome 4q23.32. Since these two disorders are caused by unrelated genetic mutations, the probability of encountering a case with concurrent PJS and hypofibrinogenemia, as in the present case, was extremely low.

Using the laboratory profile proposed by the European Network of Rare Bleeding Disorders to define the severity of an illness, the present case was classified as mild hypofibrinogenemia (15). According to the United Kingdom Hemophilia Centre Doctors' Organization guidelines, fibrinogen concentrations must be greater than 100 mg/dl to prevent surgical hemorrhage in afibrinogenemia, hypofibrinogenemia, or hemorrhagic dysfibrinogenemia. Therefore, the guideline recommends administration of the fibrinogen concentrate at a dose of 50–100 mg/kg, with smaller doses repeated if necessary at 2–4 day intervals (15). In fact, this approach is recommended as a treatment for spontaneous bleeding events as well as prophylaxis prior to any surgical intervention or against unprovoked bleeding in patients with congenital or acquired fibrinogen deficiency (16, 17). However, the optimal trough Fbg level for prophylaxis is uncertain because fibrinogen concentrate has been linked to a potential risk of thrombosis (18). In the present case, the Fbg level before EMR was 107 mg/dl. We did not know whether the bleeding in the present case was associated with hypofibrinogenemia or another cause. However, because he experienced bleeding after the EMR procedure, fresh frozen plasma or fibrinogen replacement therapy may be required before the EMR procedure for preventing further bleeding. Moreover, if the bleeding was due to hypofibrinogenemia, it may be necessary to review the fibrinogen levels for the prevention of hemorrhage as recommended by the guidelines.

PJS is a disorder characterized by the frequent development of polyps in the gastrointestinal tract as well as mole-like pigments on the skin and mucous membranes (5). The estimated prevalence of PJS ranges from 1 in 8,300 to 1 in 2,80,000 individuals, and PJS equally affects males and females (19). Polyps vary in size from a few millimeters to 7 cm. Recurrent episodes of polyp-induced intestinal obstruction and bleeding characterize the clinical course of most patients. In addition to polyposis, patients with PJS have a higher risk of gastrointestinal and extra-gastrointestinal cancers. The risk of death in patients with gastrointestinal cancer is 13 times higher than that in the general population. Moreover, such patients have a nine times higher risk of any other malignancy (particularly cancer of the reproductive organs, breast, pancreas, and lung) than the general population (20, 21). Chronic bleeding from gastrointestinal polyps can cause anemia and life-threating conditions, such as intussusception (22). Even if there are no symptoms, initial gastrointestinal surveillance should be performed at the age of approximately 8 years, and endoscopic polypectomy should be conducted for small intestinal polyps measuring ≥10–15 mm. Small-bowel surveillance is indicated in asymptomatic individuals with PJS, starting at the age of 8 years, and polypectomy is recommended for small-bowel polyps with the diameters of >15–20 mm to prevent intussusception (23). In PJS, the risk of intussusception is approximately 44% by the age of 10 years (5). Because the patient in this case was at risk of ileus due to intussusception, small-bowel surveillance for PJS should have been started at the age of 8 years.

Perforation and bleeding are common complications following endoscopy. Post-bleeding is defined as that requiring endoscopic hemostasis, with hemoglobin decreased by 2 g/dl or more, or overt bleeding before and after treatment. The reported frequency of post-bleeding ranges between 1.4% and 1.7% (24, 25). The effect of prophylactic clips on post-bleeding is controversial, but they are reported to be effective for lesions with a diameter of 20 mm or greater (26) In the present case, the resected polyps were as large as 20 and 30 mm, which could explain the postoperative bleeding. Since the patient had bleeding after EMR, it is assumed that the following bleeding prevention measures were necessary. It may have been avoided if multiple clips were applied to the wound following EMR instead of one at a time as evidenced by the fact that a 20 mm duodenal polyp resection with two clips did not bleed. Furthermore, the reason why EMR in the duodenum did not reveal post-bleeding but did in the small intestine may be attributable to the position of the polyp, but the reason for this remains unknown.

In conclusion, when polypectomy is required in patients with PJS, the physicians should consider hypofibrinogenemia one of the risk factors of postoperative bleeding, and proper prophylactic measures should be taken.

## Data Availability

The original contributions presented in the study are included in the article/Supplementary Material, further inquiries can be directed to the corresponding author/s.
